# An experimental study of simulated grant peer review: Gender differences and psychometric characteristics of proposal scores

**DOI:** 10.1371/journal.pone.0315567

**Published:** 2024-12-17

**Authors:** Karen B. Schmaling, Stephen A. Gallo

**Affiliations:** 1 Department of Psychology, Washington State University, Vancouver, Washington, United States of America; 2 Scientific Peer Advisory and Review Services, American Institute of Biological Sciences, Herndon, Virginia, United States of America; Seton Hall University, UNITED STATES OF AMERICA

## Abstract

Peer review is a decisive factor in selecting research grant proposals for funding. The usefulness of peer review depends in part on the agreement of multiple reviewers’ judgments of the same proposal, and on each reviewer’s consistency in judging proposals. Peer reviewers are also instructed to disregard characteristics that are not among the evaluation criteria. However, for example, the gender identity—of the investigator or reviewer—may be associated with differing evaluations. This experiment sought to characterize the psychometric properties of peer review among 605 experienced peer reviewers and to examine possible differences in peer review judgments based on peer reviewer and investigator gender. Participants evaluated National Institutes of Health-style primary reviewers’ overall impact statements that summarized the study’s purpose, its overall evaluation, and its strengths and weaknesses in five criterion areas: significance, approach, investigator, innovation, and environment. Evaluations were generally consistent between reviewers and within reviewers over a two-week period. However, there was less consistency in judging proposals with weaknesses. Regarding gender differences, women reviewers tended to provide more positive evaluations, and women investigators received better overall evaluations. Unsuccessful grant applicants use reviewer feedback to improve their proposals, which could be made more challenging with inconsistent reviews. Peer reviewer training and calibration could increase reviewer consistency, which is especially relevant for proposals with weaknesses according to this study’s results. Evidence of systematic differences in proposal scores based on investigator and reviewer gender may also indicate the usefulness of calibration and training. For example, peer reviewers could score practice proposals and discuss differences prior to independently scoring assigned proposals.

## Introduction

Peer review is a crucial component in the determination of grant applications that receive funding and manuscripts that are published. The importance of peer reviewers’ input is tempered by concerns about reviewers’ reliability and about reviewers’ potential lack of impartiality regarding characteristics that are not among the evaluation criteria, such as gender.

Two types of reliability are valuable in the investigation of the psychometric soundness of grant evaluation: interrater agreement, the consistency of the scores of different reviewers evaluating the same application; and test-retest reliability, the consistency of the scores of the same reviewer across time. Demonstrating reliability is important because validity presupposes reliability.

Interrater reliability has been the focus of most grant peer review reliability research. This focus is sensible because between-reviewer variability makes it less likely that an application is funded [1], and funding is needed to conduct most research and to have a successful scientific career. A recent study [2] summarized the interrater reliability reported in seven previous studies, which reported multiple-rater intraclass correlations (ICCs) of 0.00 to 0.50. However, several of these studies had used a restricted range of applications, e.g., the top 18%. The authors conducted *de novo* data analyses using the full range of applications from two different sources of peer review and found better ICCs of 0.61 and 0.64. In additional studies, ICCs of 0.41 were reported among reviewers of proposals to the Canadian [3] and Swiss [4] funding agencies, but the reliability coefficients for applications to a UK funding agency were less than 0.20 [5]. Across different funders, diverse disciplines, and different metrics of agreement, interrater reliability has ranged from zero to 0.64. No reports of grant peer reviewer test-retest reliability were found in the literature.

For many years, peer reviewers for the US National Institutes of Health (NIH) have judged proposals on five component scores—significance, innovation, investigator, approach, and environment—which informed an overall impact score. (This system is scheduled to change in 2025 [6]). Using Pearson correlations to measure the association of the six NIH scores to each other, the strongest relationship was between the overall impact score and the approach (*r* = 0.84) [7]. The scientific approach has been found to be the overall impact score-driving component in other studies [8].

Between-reviewer inconsistency could occur if some reviewers used information not among the evaluation criteria. Gender identity—both peer reviewer and investigator gender—is one potential variable associated with differences between reviewers’ scores. For example, a recent review found differences in some grant outcomes by investigator gender, such as the success rate of resubmitted proposals [9]. A Canadian study [3] found that female investigators received worse scores (.02 to .06 points lower on 1.0 (poor) to 4.9 (excellent) point scale, depending on scientific area and previous funding success) and female reviewers gave worse scores than male reviewers (.05 points lower). An Austrian study [10] found small gender differences between scores: women investigators were associated with .37 fewer points than men on a 0 (poor) to 100 (excellent) rating scale. In a US study, gender was a significant predictor of NIH overall impact scores: female investigators received better scores (.2 points lower) than men on a 1 (exceptional) to 9 (poor) scale [7]. These studies suggest that grant proposal evaluations may differ by reviewer and investigator gender.

The purpose of the present study was to characterize component scores’ association with the overall impact score; evaluate interrater and test-retest reliability; and examine the effects of reviewer and investigator gender on reviewers’ scores. We posed the following research questions: which component scores are most strongly associated with the overall scores? Do the psychometric characteristics of proposal scores achieve satisfactory levels of interrater and test-retest reliability? Is the gender of the reviewer, the investigator, or both, associated with differences in proposal scores? To investigate these questions, mock overall impact statements (OISs) for putative NIH investigator-initiated research grant (R01 mechanism) applications were used as the research materials. Participants with recent NIH-style review experience rated the OISs as if they were unassigned reviewers that provided scores based on the OIS of assigned reviewers.

## Materials and methods

The design, participants, and measures for this study were described in detail previously [11]. These methods are summarized below.

### Participants and procedure

**Fig 1:**
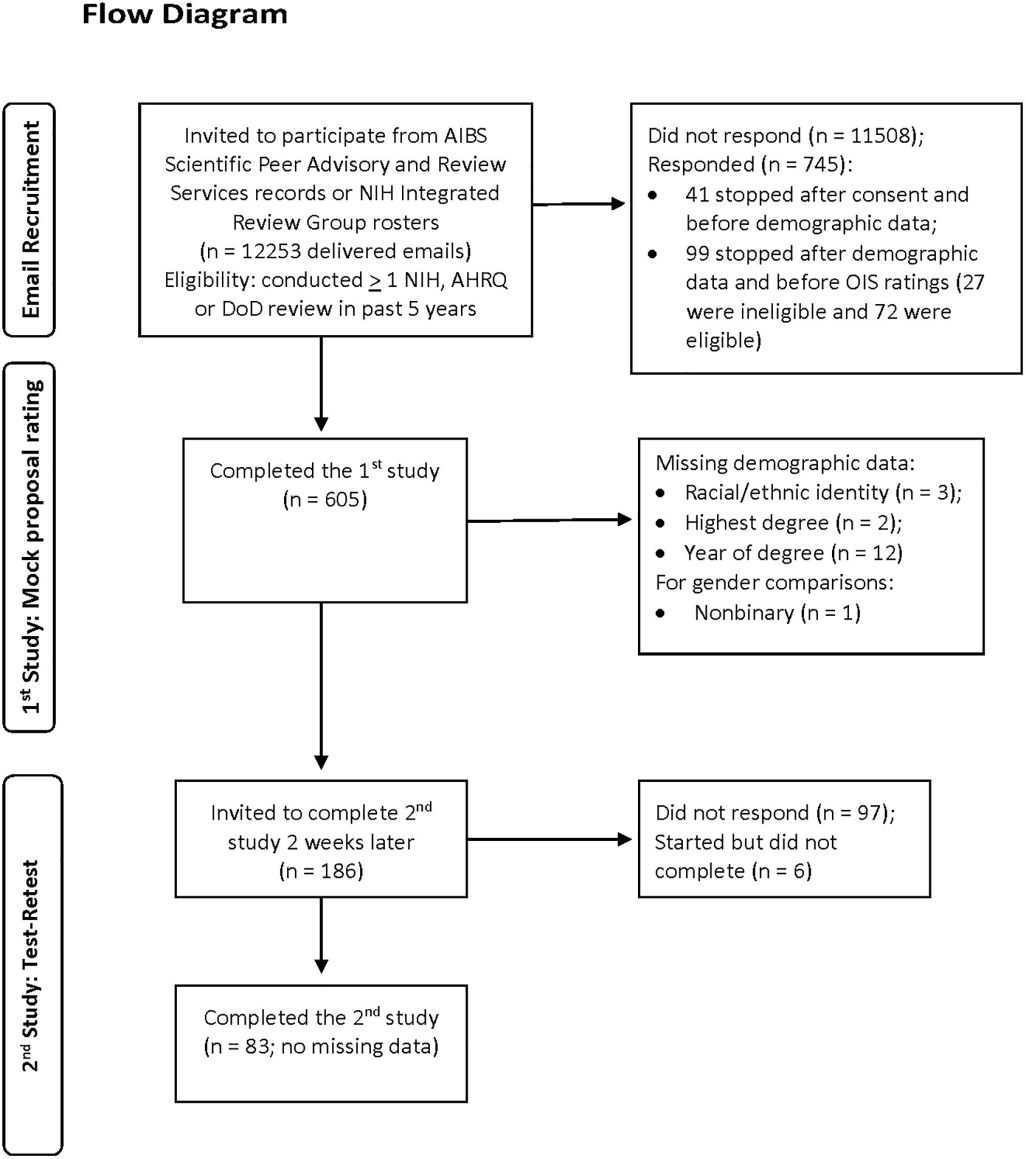
Participant recruitment and data completion flow diagram.

Participants were recruited from two sources. The first source was comprised of 10,990 people in the American Institute of Biological Science’s (AIBS) Scientific Peer Advisory and Review Services database who had reviewed for or submitted applications to one of the funders for whom AIBS had conducted peer review. The second source was comprised of 1678 scientists listed on sixteen 2020 NIH integrated review groups’ rosters. NIH reviewers’ email addresses were identified by web searches. Only scientists affiliated with US-based institutions with US-based emails were used. Between these two sources, 12,253 emails were delivered. Fig 1 is a flow diagram of the numbers of participants who were recruited with delivered emails and responded for each study; and for the first study, those who responded and were ineligible or eligible, provided incomplete or complete data, and had missing demographic data.

Participants were emailed invitations to participate with a link to complete the study on-line using Qualtrics^™^. The email described how they were identified (AIBS or NIH roster) and invited them to participate if they had done one or more NIH-style reviews in the last 5 years. One reminder invitation was sent two weeks after the initial invitation to those who had not completed the survey or opted out from further contact. 725 scientists accessed the study link and 605 scientists completed the study; (see Fig 1). The target sample size of 600 was determined by a priori power analyses based on data from a pilot study [11].

### Measures

The study components were administered in the following order, unless randomly determined as noted below.

The email invitations and the consent form described the study and inclusion criteria, which was having conducted one or more NIH-style reviews in the last 5 years. This research was deemed exempt from human subjects by the Washington State University Office of Research Assurances and granted exemption from IRB review consistent with 45 CFS 46.104(d)(2). Demographic information was collected, including degrees, year the earliest doctoral degree was earned, gender identity, English as first language, racial/ethnic identities, and the number of study sections/review committees on which the participant had served in the past five years for the NIH, Agency for Healthcare Research and Quality (AHRQ), Department of Defense (DoD), National Science Foundation (NSF), and other. If the participant reported serving on zero NIH, AHRQ, or DoD study sections, which all use similar review criteria and scoring procedures, they were told they did not meet the inclusion criteria and thanked for their interest.

Participants who met the inclusion criteria received two overall impact statements (OISs) to read and score. The process for the development and pilot testing of these OISs was described previously [11]. One OIS was the same for all participants, which we refer to herein as the control OIS. It described an outstanding proposal with no weaknesses in any of the criterion areas, and made no reference to the gender of the investigator (i.e., by name or pronouns). The other OIS was randomly assigned, which we refer to herein as the comparison OIS: the principal investigator’s gender was male or female (gender-specific names and pronouns were used). Also, the narrative suggested either no risk or weakness, or some risk in the scientific approach, the investigator, or both. There were six possible comparison OISs based on the combination of two categories of gender by three levels of risk (low PI risk and higher approach risk; higher PI risk and low approach risk; higher PI risk and higher approach risk) (S1 Fig). The order in which the participants received the two OISs was randomly determined. The randomization occurred after participants met inclusion criteria and was designed to produce balanced numbers of participants by gender, randomized OIS order (control or comparison), and randomized version of the comparison OIS. But unequal numbers of participants could result if randomized participants did not provide complete data. Anonymized study data are available on the Open Science Framework [12].

### Test-retest methods

The methods of the test-retest reliability component of the study follow. The first 186 participants who completed the first study, and who gave permission to be potentially recontacted for a future related study, were invited to complete “a similar study” two weeks after they completed the initial assessment. These participants were balanced across the six randomly chosen combinations of investigator gender and risk for the comparison OIS. Eighty-nine participants began the second study and 83 completed it (see Fig 1). The target sample size of 80 was based on an a priori power analysis from the pilot study. Each participant received the same OISs for both studies. The two assessments differed only in how the scoring options were described. The first study provided both the standard NIH numeric scale and each number’s adjectival definition, i.e., 1 = exceptional to 9 = poor, and the second study provided only adjectives without the numbers to decrease recall cueing from the first study. For example, a rating of “1/exceptional” in study one corresponded to a rating of “exceptional” in study two, which was coded with a value of 1: the pairs of ratings from each participant were used to calculate test-retest reliability.

### Statistical analysis

Descriptive statistics were used to characterize the participants, and univariate statistics were used to compare those who participated in study one with NIH reviewers; the participants in study one with study two; and men with women participants. Spearman correlations were used to examine component scores’ association with the overall impact score, separately for each OIS (control; risky approach; risky investigator; and risky approach and investigator). For interrater reliability, percent agreement (percent endorsing the modal score) was used and single-measure intraclass correlations (ICC) were calculated for absolute agreement with mixed effects (reviewer effects were random and component scores were fixed) with 95% confidence intervals for the set of six scores. ICC values of 0.00 to 0.40 are considered to represent poor agreement, 0.41 to 0.59 fair, 0.60 to 0.74 good, and 0.75 to 1.00 are considered to represent excellent agreement [13]. For test-retest reliability, single-measure ICCs were calculated between ratings over the two studies, separately by OIS, and Bland-Altman plots were used to examine if the first and second ratings were systematically biased.

The final set of analyses examined investigator and reviewer gender differences in scores, using only the 604 participants who endorsed binary gender identity. For the control OIS, Q-Q plots of scores’ distributions revealed the presence of outliers, and Levine’s test for equality of error variances showed heterogeneity in variances by reviewer gender for the innovation and approach scores. Consequently, nonparametric Mann-Whitney U tests were used to determine if women and men reviewers differed in each of the six control OIS scores. The Benjamini-Hochberg critical value was calculated to adjust for the number of tests. Effects sizes were reported based on partial eta-squared (η^2^).

For the comparison OIS scores, homogeneity of error variances (Levine’s test) and of covariance matrices (Box’s test) were inspected for violations of these assumptions: none of these tests were significant. Therefore, MANOVA was used to examine differences in comparison OIS score by reviewer gender, investigator gender, and their interaction with the control OIS score as a covariate. The covariate was used to control for reviewers’ general scoring tendencies. Demographic differences between men and women reviewers were also considered as possible covariates. Effects sizes were reported based on partial eta-squared (η^2^). All analyses were conducted using IBM SPSS V28.0 (New York, NY).

## Results

### Participants

As shown in Fig 1, complete data for the OIS ratings were provided by 605 participants for the first study, and 83 participants for the second test-retest study. The 605 participants were mostly White (74.7%; Asian 17.7%; Latinx, 3.1%; Black/African American 1.2%; Native American/Indigenous 0.2%; multiethnic 2.6%; missing 0.5%) male (63.8%: 36.0% female, 0.2% nonbinary) had only PhDs (68.8%; only MDs 15.1%; MD/PhDs 11.2%; other doctoral degrees with or without MDs and/or PhDs 4.6%; missing 0.3%) who received their first doctoral degree 29.0 (SD = 9.65) years previously and had participated on 9.97 (SD = 7.99) NIH-style panels in the past five years. Men and women participants did not differ in the number of NIH-style panels in the last five years, but participants’ first doctoral degrees had been earned more recently for women (27.92 years) than men (29.63 years, t(590) = -2.09, p = 0.037, d = -0.18), and women were more likely to have only PhD degrees (74.65%) than men (65.71%, χ^2^(1) = 5.18, p = 0.023), and less likely to identify as multiethnic or non-White than men (17.9% vs. 29%, respectively, χ^2^(1) = 9.03, p = .003).

For comparison, in 2018, men comprised 65.5% and Whites comprised 69.5% of all NIH reviewers [14]. Our sample contained slightly fewer men (~2%) and more Whites (~5%) than NIH reviewers. Years since the first doctoral degree has not been reported for NIH reviewers as far as we know, however, in 2018, 83.3% of NIH peer reviewers held the academic title of “Professor” or “Associate Professor,” indicative of substantial seniority. We could not locate peer reviewer demographic information for AHRQ or DoD, but noted that 9.3% of our sample had more peer review experience for those agencies than NIH so we cannot evaluate the similarity of their reviewers to our sample.

The subset of 83 participants who completed the second test-retest study did not differ significantly from those who completed only the first study in terms of the years since receiving their first doctoral degree, gender, PhDs only, and White ethnicity. Those who completed the second test-retest study had participated in fewer panels in the last five years (M = 8.35, SD = 7.42) than those who completed only the first study (M = 10.23, SD = 8.05, t(603) = 1.98, p = 0.048, d = 0.125).

### Interrater agreement

Table 1 shows the average and modal ratings for the overall impact score and the five criterion scores separately for each OIS. The overall impact score for the control OIS was most frequently rated a 2, corresponding to an “outstanding” proposal. The overall impact scores for the comparison OISs with risky investigator, approach, or both were most frequently rated a 2, 3, and 5, respectively, corresponding to “outstanding,” “excellent,” and “good” proposals.

**Table 1:** Average and modal scores, and percent of participants endorsing the modal score, by scoring criterion and OIS.

Score	OIS							
	Control (*N* = 605)		Risky Approach (*n* = 205)		Risky Investigator (*n* = 199)		Risky Approach & Investigator (*n* = 201)	
	*M* (SD)	% Mode (value)	*M* (SD)	% Mode (value)	*M* (SD)	% Mode (value)	*M* (SD)	% Mode (value)
Overall Impact	1.95 (0.93)	54.9 (2)	3.49 (1.35)	30.2 (3)	2.78 (1.00)	38.7 (2)	4.03 (1.35)	29.4 (5)
Significance	1.79 (1.01)	46.6 (2)	2.27 (1.23)	46.8 (2)	1.94 (0.91)	52.3 (2)	2.41 (1.15)	44.3 (2)
Innovation	2.08 (1.04)	44.1 (2)	2.33 (1.10)	43.4 (2)	2.05 (0.95)	47.7 (2)	2.50 (1.20)	44.8 (2)
Investigator	1.57 (0.96)	57.7 (1)	1.89 (1.14)	41.5(1.5)	3.93 (1.26)	**30.2 (3)**	4.20 (1.35)	**30.3 (4)**
Approach	2.26 (1.07)	50.1 (2)	4.33 (1.44)	**24.4 (5)**	2.44 (0.98)	46.2 (2)	4.73 (1.56)	**28.4 (5)**
Environment	1.53 (0.94)	60.7 (1)	1.95 (1.26)	42.9 (2)	2.49 (1.19)	34.2 (2)	3.01 (1.50)	26.4 (2)

Note: values associated with manipulated components are in bold.

Percentage agreement, calculated as the percentage of participants who rated the OIS with the modal score, was used as a measure of interrater agreement. For the control OIS, the most frequent scores were 1 or 2, with up to 61% (for environment) of participants endorsing the modal score. For the comparison OISs, the modal scores varied more between criteria, as would be expected because of the experimental manipulations. For the OIS with a risky approach, the approach was most frequently rated a 5 (25% agreement). For the OIS with a risky investigator, this criterion was most frequently rated a 3 (30% agreement). For the OIS with both a risky approach and investigator, the approach was most frequently rated a 5 (29% agreement) and the investigator was most frequently rated a 4 (31% agreement). Decreasing agreement was associated with increasing risk. The average percent of participants that endorsed the modal criterion scores in the control condition (52% agreement) was 18% greater than the average percent of participants that endorsed the modal criterion scores in the condition with both a risky investigator and approach (34% agreement).

The single-measure intraclass correlations (ICCs) follow, by OIS: control OIS, ICC = 0.614 (95% CI = 0.542, 0.675); risky approach OIS, ICC = 0.276 (95% CI = 0.139, 0.409); risky investigator OIS, ICC = 0.258 (95% CI = 0.147, 0.370); and both risky approach and investigator, ICC = 0.271 (95% CI = 0.149, 0.392).

### Associations of the component scores with the overall impact score

As shown in Table 2, approach scores had the strongest relationship with the overall impact score for all OISs.

**Table 2:** Spearman correlations of the component scores with the overall impact score, by OIS.

Score	Control OIS	Comparison OISs		
		Risky Approach	Risky Investigator	Risky Approach and Investigator
Significance	0.65	0.49	0.49	0.48
Innovation	0.60	0.28	0.29	0.34
Investigator	0.48	0.30	**0.45**	**0.44**
Approach	0.66	**0.70**	0.50	**0.70**
Environment	0.43	0.23	0.34	0.31

Note: Coefficients in bold font are associated with the manipulated components (approach, investigator, or both).

### Test-retest reliability

Across the six scores, the average ICC for the control OIS was 0.52, and for the comparison OISs it was 0.31 for risky approach, 0.33 for risky investigator, and 0.47 for risky approach and investigator. Table 3 shows the test-retest ICCs separately for each score and each OIS. Environment tended to be rated inconsistently. There was more variability in reviewers’ test-retest consistency for the comparison OISs than for the control OIS, and among the comparison OISs, single sources of risk were rated more inconsistently than were two sources of risk.

**Table 3:** Test-retest intraclass correlations, by OIS.

Criterion Score	OIS			
	Control (*n* = 83)	Risky Approach (*n* = 26)	Risky Investigator (*n* = 27)	Risky Approach & Investigator (*n* = 30)
Overall Impact	.61	.00	.53	.62
Significance	.46	.46	.07	.45
Innovation	.65	.45	.28	.41
Investigator	.51	.52	**.52**	**.71**
Approach	.54	**.11**	.55	**.47**
Environment	.35	.29	.00	.15

Note: coefficients associated with manipulated components are in bold.

Visual inspection of the Bland-Altman plots of the difference in a reviewer’s score versus their average score revealed no evidence of systematic test-retest bias for the overall impact score for both the control and comparison OIS (see S2 Fig). The visual impressions were supported by statistical tests of the relationship between with difference and average scores, which were not significant.

### Gender comparisons

Mann-Whitney U tests were used to examine differences in scores based on reviewer gender for each component of the control OIS, using (see Table 4). According to Benjamini-Hochberg critical values, there were significant differences between women and men reviewers for all criterion scores except environment. In all cases, women reviewers gave significantly lower (better) scores than men reviewers. The magnitude of this difference was 11% for the overall impact score.

**Table 4:** Differences in control OIS criterion scores by reviewer gender.

Criterion Score	Mann-Whitney U	p	Effect size (η^2^)	M (SD)	
				Women (n = 218)	Men (n = 386)
Overall Impact	37171.00	.008	.012	1.81 (0.81)	2.03 (0.99)
Significance	36730.50	.004	.014	1.65 (0.86)	1.88 (1.08)
Innovation	36740.00	.006	.013	1.91 (0.85)	2.18 (1.13)
Investigator	36986.50	.005	.013	1.45 (0.87)	1.63 (1.00)
Approach	36086.50	.002	.016	2.08 (0.93)	2.37 (1.13)
Environment	40705.00	.441	.001	1.48 (0.84)	1.56 (1.00)

Next, MANOVA was used to examine differences in comparison OIS score by reviewer gender, investigator gender, and their interaction, with the control OIS score as a covariate. (The control OIS score had a significant effect but none of the three reviewer characteristics that differed between men and women reviewers—years since first doctoral degrees, having only PhD degrees, identifying as White only—had significant multivariate effects: they were omitted from the analyses reported here.) Multivariate tests found significant main effects for reviewer gender (F(6, 595) = 2.41, p = .026, η^2^ = .024) and investigator gender (F(6,595) = 2.16, p = .046, η^2^ = .021), but the reviewer by investigator gender interaction was not significant (F(6,595) = 0.80, p = .568, η^2^ = .008). Table 5 shows the results by score. Innovation differed significantly by reviewer gender: women reviewers gave lower scores than men reviewers: estimated marginal means = 2.12 and 2.39, respectively. Women investigators received lower (better) overall scores than men investigators: estimated marginal means = 3.28 and 3.57, respectively.

**Table 5:** Comparison OIS criterion scores by reviewer and investigator gender, controlling for control OIS criterion scores.

Criterion Score	Factor		
	Reviewer Gender *F*(1,596) (*p*) (η^2^)	Investigator Gender *F*(1,596) (*p*) (η^2^)	Reviewer x Investigator Gender *F*(1,596) (*p*) (η^2^)
Overall Impact	0.05 (.833) (.000)	**6.21 (.013) (.010)**	0.31 (.577) (.001)
Significance	2.78 (.096) (.005)	0.74 (.389) (.001)	0.24 (.628) (.000)
Innovation	**8.83 (.003) (.015)**	1.22 (.271) (.002)	0.03 (.870) (.000)
Investigator	0.41 (.524) (.001)	1.50 (.222) (.002)	0.53 (.466) (.001)
Approach	0.28 (.600) (.000)	0.11 (.739) (.000)	0.04 (.846) (.000)
Environment	0.20 (.655) (.000)	0.03 (.855) (.000)	0.98 (.323) (.002)

Note: statistically significant effects in bold font

## Discussion

This study found that peer reviewers’ reliability was moderate overall, whether it was examined as agreement with the modal score or test-retest reliability after a two-week delay. However, reliability decreased when the proposal had areas of weakness. For example, the proportion of reviewers who used the modal score for the control OIS was halved for the manipulated criteria (approach, investigator, or both) in the comparison OIS.

In terms of associations among the component scores, this study reinforces previous studies’ results [7, 8]: of the five components of the NIH review criteria, the approach score had the strongest association with the overall impact score. Furthermore, although the descriptions of significance, innovation, and environment were the same for all of the comparison OISs, ratings of these criteria seemed to have been negatively influenced, or contaminated, by weaknesses in the approach and/or the investigator. While speculative, this pattern of results was consistent with negativity bias [15]: weaknesses were more influential and salient than strengths. The test-retest data also reflected the salience of weaknesses: test-retest agreement was greater for the components that were manipulated in the comparison OISs—risky investigator and/or approach—than test-retest agreement for these components in the control OIS. In other words, reviewers may have remembered weaknesses more than strengths and scored the former more reliably [16]. We were unable to validate this potential interpretation by, for example, follow-up interviews with the participants. Such work remains to be done in future research.

This study adds to the extant literature on peer reviewers’ reliability. Reviewers were relatively consistent with each other on par with previous studies [2–5]. Reviewers were also consistent with themselves over a two-week period. However, better reliability was observed for an outstanding application than one with areas of relative weakness. Given that research funding is highly competitive, it is arguably most important that reviewers reliably identify outstanding proposals—which they did in this study. If more research funding was to become available, the reliable identification of good and very good proposals that might qualify for funding would become more essential. Improved reviewer reliability could also lead to more consistent feedback to investigators. Investigators can be frustrated with inconsistent or contradictory feedback [17]. Resubmission is often required for success and responsiveness to previous reviews is an important component in the evaluation of resubmitted proposals. Reviewer training videos and calibration discussions are intended to enhance reliability, but do not do so consistently [18, 19]. Further work along these lines would be valuable.

This study also examined differences in scores based on reviewer and investigator gender. For the control OIS, women peer reviewers gave significantly better scores to all criteria except environment than did men peer reviewers, accounting for small amounts (1%) of the variance in scores. For the comparison OIS (with control OIS scores as covariates), women reviewers gave better innovation scores than did men reviewers, accounting for 2% of the variance in scores. These results are consistent with some previous studies that found women reviewers to give better scores than men [7], for example in the sciences [20]. But they are inconsistent with other studies that found women reviewers to give worse scores than men reviewers [3, 10, 21]. We do not know why women reviewers gave better scores, which could be due to more positive or less critical mindsets, desire to nurture other scientists, or other factors. But as there is no gold standard, we cannot determine who is accurate, and who is biased.

Our study also found that investigators’ gender accounted for about 2% of the variance in scores for proposals with weaknesses. The effect sizes and gender differences in scores may seem small, but small differences can result in unfunded or funded proposals. For example, among 2023 R01 applications to the National Institute of General Medical Sciences, the likelihood of funding decreased with scores at approximately the 22nd percentile and above (overall impact scores are converted to percentiles for each review panel) (see Fig 4 in reference [22]). Reviewers gave better overall impact scores to women investigators than men, contrary to studies that have found women to receive worse scores [3, 10]. However, no differences by investigator gender were revealed for the other criteria. We offer two speculative reasons for the gender difference: First, participants may have guessed that investigator gender was manipulated in the study and wished to appear to be fair. But only three (0.5%) participants left comments that referenced gender in the study. A woman’s name and pronouns may cue positive bias [23] to attempt to counteract historical discrimination against women scientists, or to mitigate concerns about appearing sexist. Finally, there were no interactions between reviewer and investigator gender for the overall or component scores, suggesting a lack of gender affinity bias—a preference for others of the same gender.

### Limitations

Although a minority of the invited reviewers participated, the characteristics of those who did so were similar to NIH reviewers at the time. More recently, however, NIH reviewers have included fewer men and Whites: these changes in NIH reviewer demographics means that our sample reflects current panels’ demography less well than the panels of several years ago. This study was modeled on the role of unassigned reviewers in NIH study sections, who must score a proposal they have not read based on the reviews of the assigned reviewers. In actual panels, unassigned reviewers’ scores are very similar (0.06 points greater) to assigned reviewers’ scores [24]. In this study, however, unlike actual NIH study sections, no meetings and discussions occurred that would have also informed the reviewers’ scores. No gender was specified for the investigator of the proposal without weaknesses, the control OIS, so we cannot know if women investigators would have been evaluated more positively than men, as they were for proposals with weaknesses. Furthermore, the basic science in the OISs could have been interpreted as research that women scientists do, possibly leading to more positive evaluations of the control OIS by women reviewers, and of better overall scores for women investigators of the comparison OIS. This possibility is tempered by evidence that both men and women MDs and life science PhDs have stronger implicit and explicit associations of men with science than of women [25].

## Conclusions

Experienced grant peer reviewers are relatively reliable with each other and with themselves over a two-week period when they evaluate a proposal without weaknesses. Women reviewers gave this strong proposal better scores than did men reviewers. Peer reviewer reliability suffers, however, when evaluating proposals with weaknesses. In proposals with weaknesses, women reviewers continued to give better scores than men, but only in one area: innovation. Furthermore, proposals from women investigators were scored more favorably, but only the overall impact score: no investigator gender differences were found for scores on the other criteria (significance, innovation, investigator, approach, environment). Further research to determine the sources of and rationales for these inconsistencies would be valuable. Gender differences in reviewers’ scores for the same OIS may also suggest the need for calibration, as do differences in scores by investigator gender. Although gold standards for “correct” scores do not exist, peer reviewers could score practice OISs and discuss differences prior to independently scoring new proposals. And—if replicated—be mindful of any tendencies to evaluate proposals differently based on gender identity.

## Supporting information

S1 Fig Design for the gender comparisons. (DOCX)

S2 Fig Bland-Altman plots for the control and comparison OISs. (DOCX)
