# Supplementary material for: An experimental study of simulated grant peer review: Gender differences and psychometric characteristics of proposal scores
Source: PLoS One. 2024 Dec 17;19(12):e0315567. doi: 10.1371/journal.pone.0315567 (PMC11651561; doi:10.1371/journal.pone.0315567)
Supplement: S1 Fig — (DOCX) [file pone.0315567.s001.docx]

**S1 Fig. Design for the gender comparisons.**

**Design for the control OIS**

| Control OIS |  | Women reviewers |
| --- | --- | --- |
|  |  | Men reviewers |

**Design for the comparison OISs**

| Risky approach only |  | Women reviewers |  | Women investigators |
| --- | --- | --- | --- | --- |
|  |  |  |  | Men investigators |
|  |  | Men reviewers |  | Women investigators |
|  |  |  |  | Men investigators |
| Risky investigator only |  | Women reviewers |  | Women investigators |
|  |  |  |  | Men investigators |
|  |  | Men reviewers |  | Women investigators |
|  |  |  |  | Men investigators |
| Risky approach and investigator |  | Women reviewers |  | Women investigators |
|  |  |  |  | Men investigators |
|  |  | Men reviewers |  | Women investigators |
|  |  |  |  | Men investigators |
