# Supplementary material for: An experimental study of simulated grant peer review: Gender differences and psychometric characteristics of proposal scores
Source: PLoS One. 2024 Dec 17;19(12):e0315567. doi: 10.1371/journal.pone.0315567 (PMC11651561; doi:10.1371/journal.pone.0315567)

**S2 Fig. Bland-Altman plots for the control and comparison OISs.**

**Bland-Altman plot for the control OIS**

Bland-Altman plot of the average (X axis) and difference (Y axis) of the overall impact scores from the 1^st^ and 2^nd^ evaluations of the control OIS. The red line is the average difference (M = -.048) and the green lines denote the 95% CIs around the average. The size of the plot points reflects the number of participants with those values. The relationship between the average and difference scores was R = .063, F (1,81) = .321, p = .573.


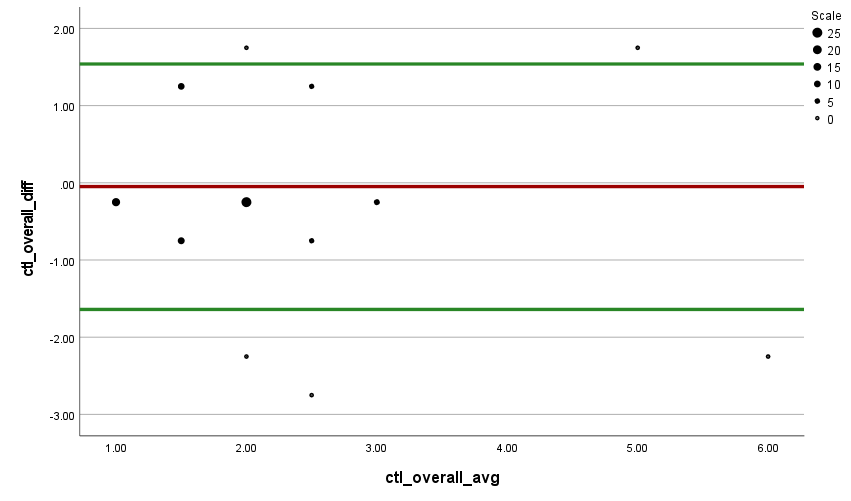


**Bland-Altman plot for the comparison OIS**

Bland-Altman plot of the average (X axis) and difference (Y axis) of the overall impact scores from the 1^st^ and 2^nd^ evaluations of the comparison OIS. The red line is the average difference (M = -.06) and the green lines denote the 95% CIs around the average. The size of the plot points reflects the number of participants with those values. The relationship between the average and difference scores was R = .078, F (1,81) = .490, p = .486.


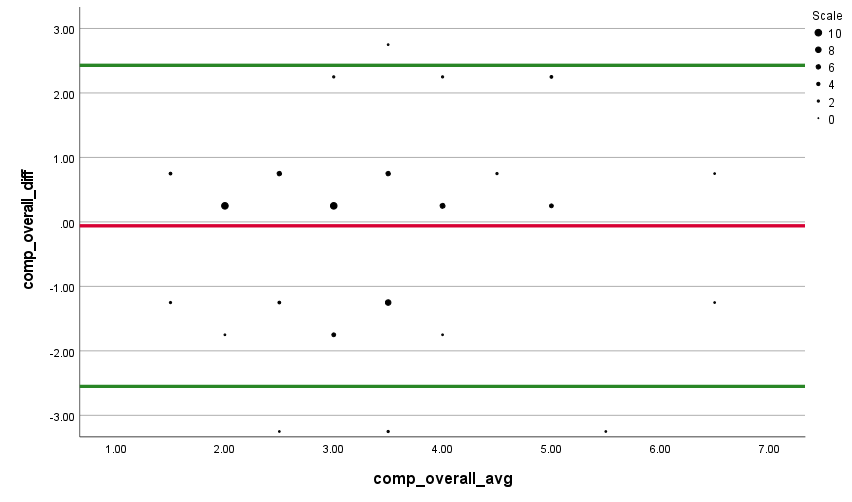

Supplement: S2 Fig — (DOCX) [file pone.0315567.s002.docx]
